# Supplementary material for: ODGI: understanding pangenome graphs
Source: Bioinformatics. 2022 May 13;38(13):3319–26. doi: 10.1093/bioinformatics/btac308 (PMC9237687; doi:10.1093/bioinformatics/btac308)
Supplement: btac308_Supplementary_Data [file btac308_supplementary_data.pdf]

S Supplement

S.1 Graph topology visualization

In an *odgi viz* visualization, given a path X traversing two nodes A and B, the corresponding edge is represented by a black line starting from the left or right of node A if this node is traversed in reverse or forward, respectively, by path X; the black line ends to the left or right of node B if this node is traversed in forward or reverse, respectively, by path X. Consequently, if two consecutive nodes are linked both in forward, no edge is shown (it would be 0 pixels long, as it would start from the right of the first node and ends to the left of the second one).

S.2 Graph navigation and untangling

Pangenome graphs can model alignments of many genomes. With *odgi untangle*, users can extract pairwise alignment information between a given set of “query” sequences and a given set of “target” sequences (used as references). While pangenome graphs may contain looping structures that imply many-to-many alignments between the pangenome sequences, these untangled alignments map each segment of the queries to a single segment in the set of targets. *odgi untangle* first discovers segment boundaries using standard approaches for detecting repeats in sequence graphs (Pevzner, 2004). We finally “untangle” by finding the target segment that best matches each query segment using the *path jaccard* context mapping model. Moreover, to obtain base-level precise information on the relationships between the repeated sequences, we can align them by using the pairs of regions that came from the untangling to guide the alignment (Guarracino *et al.*, 2021).

odgi tips can identify the break point positions of the contigs relative to the reference(s) in the graph by walking from the ends of each contig until a reference node is found. It could be that the reference visits the node several times. Therefore, for each contig range (a tip) *odgi tips* takes a look at each possible reference window and finds the most similar one using the *path jaccard* concept. The output is a BED file with the best reference hit and position for each of the contigs’ ends.

S.2.1 The GFF liftover

A GFF file contains annotations for one or more paths in the graph. For each annotation, we know the start and end within that path. So we can annotate all nodes that are visited by such a path range with the information from the attribute field. If there are overlapping features, we append the annotation for each node. Using the same coloring schema as in *odgi viz* we generate a color for each annotated node by its collected annotation.

If a subgraph was as a result from e.g. *odgi extract*, the path names are usually in the form of name:start-end. *odgi position* is able to automatically detect this and adjust the positions given in the GFF on the fly to the new positions given in the subgraph. For each GFF entry, it just subtracts the “missing” number of nucleotides from the start and end field. That’s how we adjust for the subgraph annotation.

S.2.2 Path Jaccard concept

odgi tips, *odgi untangle*, and *odgi position* all translate a query path position to a target path position. In a repeat region, it could be that the node of the query path position (N_q) is visited several times by the target path. We use the graph *path jaccard* concept to infer the best target position for a given query path position. For the query position we look at each possible target window and find the most-similar one:

1. Starting from N_q we follow the steps of one target path position a certain nucleotide distance to the left and a certain nucleotide distance to the right, also called context. The ODGI default distance is 10kbp for each direction.
2. We collect all node identifiers that are visited this way in a multi-set.
3. Repeat the same process when following the query path position steps.

Now we have two multi-sets of node identifiers, the target path position T one, and the query path position Q one. We apply the Jaccard measure to estimate the similarity between these two multi-sets (Equ. 1).

$$J(T, Q) = \frac{|T \cap Q|}{|T \cup Q|} \quad (1)$$

The "|" indicate that, after we joined our sets together, we actually calculate the total length in nucleotides represented by the intersection node set in the nominator, and by the union node set in the denominator, respectively. The division of the two nucleotide lengths then gives us the jaccard measure for the two path positions. We collect these jaccard measurements for all possible target path positions. The largest *path jaccard* determines the actual target path position for the translation.

Dependent on how repeat-streaked the graph is and how it was constructed, one might want to adjust context size, to get an even more precise positional translation. If we can’t follow the full distance to one direction, because we are at an end of a sequence, we also adjust this for all other context evaluating windows.

In the following an example which is based on the graph in Figure S1. Let’s assume, we want to translate the query path position *query:3* to

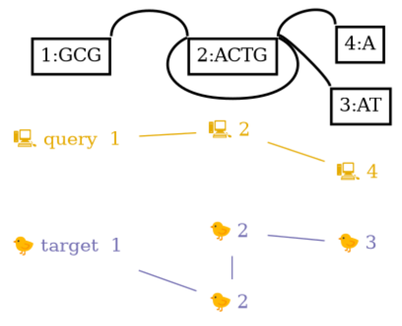

Fig. S1: Simple example graph to demonstrate the *path jaccard* concept. Generated with *vg view* and *graphviz’ dot* command. On top are the nodes connected with edges. On the bottom the paths through the nodes. Each path has a different color and emoji.

a path position in path *target* with a context distance of 2 nucleotides per direction. Node *2* is where *query:3* is located. The node is traversed two times by *target*. Therefore, we apply the *path jaccard* concept to do a precise position translation. Starting from node *2*, we collect nodes $\{1, 2, 4\}$ for path *query*. For path *target* we have two possible steps to start our collection from: The first or the second step in node *2*. We obtain the node sets $T_1 = \{1, 2, 2\}$ and $T_2 = \{2, 2, 3\}$.

$$J_1(T_1, Q) = \frac{|T_1 \cap Q|}{|T_1 \cup Q|} = \frac{|\{1,2,2\} \cap \{1,2,4\}|}{|\{1,2,2\} \cup \{1,2,4\}|} = \frac{|\{1,2\}|}{|\{1,2,2,4\}|} \widehat{=} \frac{3+4}{3+4+4+1} \approx 0.58 \quad (2)$$

$$J_2(T_2, Q) = \frac{|T_2 \cap Q|}{|T_2 \cup Q|} = \frac{|\{2,2,3\} \cap \{1,2,4\}|}{|\{2,2,3\} \cup \{1,2,4\}|} = \frac{|\{2\}|}{|\{1,2,2,3,4\}|} \widehat{=} \frac{4}{3+4+4+2+1} \approx 0.29 \quad (3)$$

We observe a *path jaccard* of 0.58 for T_1/Q (Equ. 2) and 0.29 for T_2/Q (Equ. 3). Therefore, we translate query path position *query:3* to the target path position *target:3*.

S.3 Editing

Subgraphs can be extracted by using the paths in the graph as coordinate systems to guide the process. For such operation, *odgi extract* allows users to extract specific regions of the graph as defined by query criteria. Regions of interest can be specified by graph nodes or path range(s), also in BED format. Furthermore, it is possible to indicate a list of paths to be preserved completely in the extracted graph. We begin by collecting all graph nodes that fall within the ranges to extract (and the paths to preserve, if requested). Users can specify the number of steps or nucleotides to expand the selection and include neighboring nodes. Then, edges connecting all selected nodes are added in the subgraph under construction. Finally, the portions of the paths (i.e., the subpaths) walking through the selected nodes are extracted and added to the new subgraph. Subpaths are searched in parallel, created serially, and extended in parallel again thanks to the parallelism enabled by the ODGI data structure (see §4.1), making *odgi extract* a scalable solution to extract also complex subregions presenting nodes with very high node depth.

Pangenome graphs can embed multiple chromosomes as separated connected components (inter-chromosomal structural variants would join the components into bigger ones). *odgi explode* separates the connected components in different ODGI format files, while *odgi squeeze* allows merging multiple graphs into the same ODGI format file, preventing node ID collisions.

Pangenome graphs can be used in a variety of applications, ranging from read mapping to variant identification and genotyping (Eizenga *et al.*, 2020b). However, graphs presenting complex topology can increase the computational overhead of many downstream analyses. ODGI offers multiple commonly-needed basic operations on the topology of the graph and its nodes.

For simplifying the graph structure, users can use *odgi prune* to take away complex parts as defined by query criteria, while with *odgi break* they can remove cycles in the graph, reducing the complexity of the graph topology. Furthermore, *odgi groom* allows removing spurious inverting links by exploring the graph from the orientation supported by most paths; the process does not remove any genetic information, but only edits how the sequences are represented in the graph.

To enable efficient sequence alignment against the graph, long nodes can be divided into shorter nodes at a maximum requested size using *odgi chop*. Partial order alignment, which consists of aligning sequences against a directed acyclic graph (DAG), is frequently used in pangenome building pipelines (Garrison *et al.*, 2021), but the current implementations return DAGs with 1-bp long nodes. *odgi unchop* allows joining nodes that can be merged without changing the graph topology, nor the embedded sequences, obtaining an equivalent, but more compact, representation of the graph.

S.4 Sorting and node identifier compaction

Most subcommands in ODGI require and verify that the input graph's node identifiers (IDs) are optimized, that is compacted from 1 to N where N is the number of nodes in the graph. If this assumption is violated, *odgi sort* provides functionality to optimize the graph. This means that the first node identifier (ID) starts at 1 and the last node ID is the number of nodes. All sorting operations update the graph in place with an efficient ID rewriting algorithm. The graph is then updated in place. First, the node identifiers are normalized (from 1 to number of nodes) including the adjustment of the edges. Second, path information, including both path metadata that points into the start and end steps of the path, plus each step of every path, is updated, too. We point out that changing the node order does not change our coordinate systems based on paths. These will now refer to a new node ordering.

When we sort a graph, we switch the node IDs of the nodes according to the result of the sorting algorithm. For example, if a random sort was applied, all existing node IDs would be replaced with new, random ones (the largest node ID would still correspond to the number of nodes on the graph). We would update the edge and path information as described in the paragraph above. The reordering of nodes has a great influence on how the pangenome looks like (Fig. S2).

S.5 Linear projections

Pangenome graph topology can be derived by applying *odgi matrix*, obtaining information on graph nodes connections in textual sparse matrix format. To investigate on the genomic sequences encoded in the graph, *odgi paths* allows users to calculate pairwise overlap statistics of groupings of paths and emit all path sequences in FASTA format, and it also allows the generation of a "pangenome matrix" that reports the copy number (presence/absence) of each path over each node.

In standard practice, pangenome analysis examines presence-absence variations (PAVs), which correspond to loci that are present in some samples but not others. *odgi pav* uses a set of genomic intervals (in BED expressed in the coordinate space of paths in the graph) to demarcate PAVs. It then describes the coverage of all paths relative to these PAVs, yielding matrix or tabular representation. The precise determination of PAVs based on graph topology remains an open problem, but practically any method capable of generating a BED file can be used here. This lets us define PAVs using repeat or gene annotations of the genomes represented as paths in the graph. A simple technique is to take the output of *odgi flatten*, which generates a linearization of the graph by emitting the pangenome sequence (the concatenation of all node sequences) in FASTA format, and the projection of all paths on the linearized sequence in BED format relative to the graph's paths.

ODGI also supports an experimental binned representation of graphs designed to support the study and visualization of pangenomes at different scales of resolution. *odgi bin* summarizes graph path information into bins of a specified size, generating a summarized view of gigabase scale graphs in TSV or JSON file formats. We have further supported this binning approach in pangenome graph ontology model (Yokoyama *et al.*, 2020).

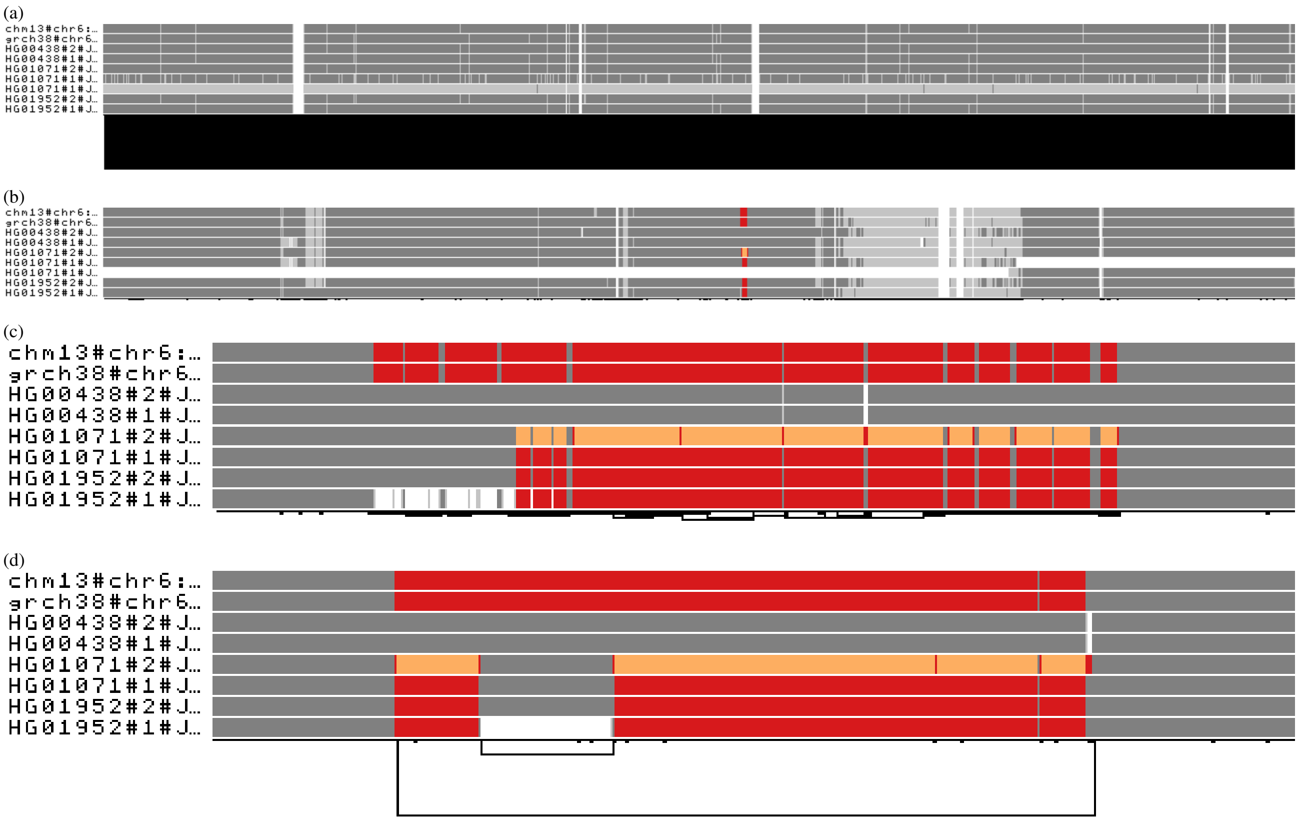

Fig. S2: Sorting and visualizing the human major histocompatibility complex (MHC) and complement component 4 (C4) pangenome graphs. All visualization are made with *odgi viz* by coloring the paths by node depth: using the Spectra color palette with 4 levels of node depths, white indicates no depth, while grey, red, and yellow indicate depth 1, 2, and greater than or equal to 3, respectively. **(a)** Visualization of a MHC pangenome graph with nodes randomly sorted from left to right. The image shows a region 5 Mbp-long. The bad node order completely hides the linear graph structure. **(b)** Visualization of the same MHC pangenome graph but sorted by applying the path-guided stochastic gradient descent algorithm (PG-SGD). The graph globally shows a linear structure, without long range links (at the bottom of the image). Furthermore, the C4 region is visible as a region with red and orange paths. **(c)** Visualization of the PG-SGD sorted C4 subgraph. The image shows a region 83361 bp-long. The C4 region is still not well sorted locally, with the node order that still hides the underlying copy number variation status present in such a region. **(d)** Visualization of the same C4 pangenome graph but sorted by applying a topological sorting. The graph shows its structure: the two references present two different allele copies of the C4 genes (in red), both of them including the HERV sequence. HG01071#2 presents 3 copies of the *locus* (orange), of which one contains the HERV sequence (gray in the middle of the orange). In HG01952#1, the HERV sequence is absent (white in the middle of the red).

S.6 Evaluation

Table S1: Performance measurements when transforming a human chromosome 6 pangenome graph into the tool's native format. **haps** is the number of haplotypes in the graph. Displayed are the mean results after 10 runs.

		time in seconds		memory in gigabytes	
threads	haps	odgi build	vg convert	odgi build	vg convert
1	1	**10.78**	21.92	1.15	**0.56**
1	2	**14.55**	28.71	1.48	**0.78**
1	4	**22.28**	45.02	1.82	**1.51**
1	8	**43.41**	78.67	**2.52**	3.01
1	16	**76.60**	133.27	**3.57**	5.54
1	32	**176.55**	292.32	**5.90**	11.29
1	64	**322.50**	497.51	**10.19**	21.3
2	1	**10.56**	21.18	1.15	**0.56**
2	2	**12.79**	27.51	1.48	**0.78**
2	4	**17.08**	43.64	1.81	**1.51**
2	8	**28.90**	75.52	**2.42**	3.01
2	16	**47.93**	126.71	**3.46**	5.54
2	32	**102.67**	280.12	**5.58**	11.29
2	64	**171.31**	457.37	**9.52**	21.3
4	1	**10.54**	20.69	1.15	**0.56**
4	2	**12.63**	27.90	1.48	**0.78**
4	4	**14.80**	42.36	1.88	**1.51**
4	8	**21.00**	74.80	**2.18**	3.01
4	16	**32.69**	123.41	**3.08**	5.54
4	32	**65.30**	271.10	**4.87**	11.29
4	64	**105.51**	451.54	**8.70**	21.3
8	1	**11.53**	20.33	1.15	**0.56**
8	2	**13.16**	27.18	1.48	**0.78**
8	4	**15.61**	41.77	1.85	**1.51**
8	8	**19.63**	73.09	**2.20**	3.01
8	16	**28.43**	131.82	**2.87**	5.54
8	32	**46.23**	256.74	**4.30**	11.29
8	64	**71.55**	441.59	**6.91**	21.30
16	1	**11.18**	21.14	1.15	**0.56**
16	2	**13.27**	26.74	1.48	**0.78**
16	4	**15.55**	41.90	1.87	**1.51**
16	8	**21.16**	73.36	**2.24**	3.01
16	16	**29.09**	136.96	**2.92**	5.54
16	32	**46.58**	269.61	**4.36**	11.29
16	64	**70.84**	442.71	**6.89**	21.30

Table S2: Performance measurements when extracting the centromeric region of a human chromosome 6 pangenome graph. **haps** is the number of haplotypes in the graph. Displayed are the mean results after 10 runs.

		time in seconds		memory in gigabytes	
threads	haps	odgi extract	vg chunk	odgi extract	vg chunk
1	1	3.65	**3.34**	1.07	**0.42**
1	2	**6.08**	14.25	1.22	**0.95**
1	4	**15.96**	58.67	**1.51**	3.06
1	8	**37.16**	170.63	**1.91**	8.12
1	16	**67.47**	364.98	**2.49**	15.25
1	32	**161.32**	968.71	**4.00**	33.65
1	64	**313.05**	1897.00	**6.96**	60.37
2	1	3.67	**3.33**	1.07	**0.42**
2	2	**5.50**	13.57	1.22	**0.96**
2	4	**12.33**	56.44	**1.51**	3.06
2	8	**24.06**	170.62	**1.92**	8.12
2	16	**43.49**	376.67	**2.51**	15.25
2	32	**97.03**	987.85	**4.03**	33.65
2	64	**187.51**	1907.98	**7.00**	60.37
4	1	3.64	**3.32**	1.07	**0.42**
4	2	**5.59**	13.40	1.22	**0.96**
4	4	**9.93**	57.67	**1.52**	3.06
4	8	**16.58**	174.94	**1.94**	8.11
4	16	**27.99**	374.99	**2.52**	15.26
4	32	**56.94**	992.85	**4.06**	33.65
4	64	**108.34**	1885.91	**7.04**	60.37
8	1	3.61	**3.30**	1.07	**0.42**
8	2	**5.47**	14.01	1.22	**0.96**
8	4	**8.43**	58.69	**1.53**	3.06
8	8	**12.40**	180.51	**1.98**	8.11
8	16	**19.11**	379.25	**2.55**	15.26
8	32	**36.35**	991.09	**4.11**	33.65
8	64	**68.54**	1841.57	**7.11**	60.37
16	1	3.64	**3.34**	1.07	**0.42**
16	2	**5.53**	14.67	1.22	**0.95**
16	4	**7.62**	58.22	**1.54**	3.06
16	8	**10.55**	171.90	**2.03**	8.12
16	16	**14.80**	373.70	**2.60**	15.26
16	32	**25.51**	981.79	**4.27**	33.65
16	64	**46.87**	1838.51	**7.23**	60.37

Table S3: Performance measurements when visualizing a human chromosome 6 pangenome graph. **haps** is the number of haplotypes in the graph. Both *odgi viz* and *vg viz* were run with 1 thread. Displayed are the mean results after 10 runs. *A 816MB SVG was produced which can't be opened by any program. **All produced SVGs were empty except for an XML header.

	time in seconds			memory in gigabytes		
**haps**	**odgi viz**	**vg viz**		**odgi viz**	**vg viz**	
1	**4.14**	43.40	*	**1.04**	7.09	*
2	**5.30**	57.94	**	**1.14**	9.69	**
4	**7.85**	76.15	**	**1.27**	14.05	**
8	**12.95**	109.16	**	**1.53**	21.84	**
16	**22.33**	170.72	**	**1.88**	36.97	**
32	**49.24**	303.40	**	**2.87**	69.88	**
64	**102.82**	543.50	**	**4.78**	127.36	**

Table S4: Performance measurements when locating all path positions of a node in a human chromosome 6 pangenome graph. **haps** is the number of haplotypes in the graph. Displayed are the mean results after 10 runs. The number of threads does not affect the running time or memory consumption. *vg find* had to be run for each path position. The total run time of finding all path positions of a node is reported here.

		time in seconds		memory in gigabytes	
**threads**	**haps**	**odgi position**	**vg find**	**odgi position**	**vg find**
1	1	2.58	**0.16**	1.01	**0.21**
1	2	3.19	**0.39**	1.11	**0.26**
1	4	3.52	**1.23**	1.23	**0.42**
1	8	**3.97**	4.28	1.49	**0.74**
1	16	**5.07**	14.32	1.81	**1.25**
1	32	**7.79**	55.61	2.73	**2.46**
1	64	**15.60**	198.54	**4.51**	4.56
2	1	2.54	**0.16**	1.01	**0.21**
2	2	3.17	**0.38**	1.11	**0.26**
2	4	3.55	**1.24**	1.23	**0.42**
2	8	**3.95**	4.31	1.49	**0.74**
2	16	**5.08**	14.10	1.81	**1.25**
2	32	**7.76**	55.56	2.73	**2.46**
2	64	**15.34**	198.56	**4.51**	4.56
4	1	2.67	**0.16**	1.01	**0.21**
4	2	3.15	**0.38**	1.11	**0.26**
4	4	3.55	**1.23**	1.23	**0.42**
4	8	**3.98**	4.31	1.49	**0.74**
4	16	**5.06**	14.23	1.81	**1.25**
4	32	**7.79**	55.66	2.73	**2.46**
4	64	**15.41**	196.6	**4.51**	4.56
8	1	2.59	**0.16**	1.01	**0.21**
8	2	3.20	**0.38**	1.11	**0.26**
8	4	3.55	**1.25**	1.23	**0.42**
8	8	**3.99**	4.31	1.49	**0.74**
8	16	**5.07**	14.20	1.81	**1.25**
8	32	**7.70**	55.12	2.73	**2.46**
8	64	**15.9**	203.02	**4.51**	4.56
16	1	2.62	**0.16**	1.01	**0.21**
16	2	3.16	**0.38**	1.11	**0.26**
16	4	3.55	**1.23**	1.23	**0.42**
16	8	**3.95**	4.26	1.49	**0.74**
16	16	**5.1**	14.3	1.81	**1.25**
16	32	**7.66**	54.78	2.73	**2.46**
16	64	**15.5**	202.57	**4.51**	4.56

Table S5: Disk space measurements of GFAv1 and the respective tools' binary formats. **haps** is the number of haplotypes in the graph. For VG, the file size of the XG format was measured.

	disk space in megabytes		
**haps**	**GFAv1**	**ODGI**	**VG**
1	283	604	**189**
2	333	660	**237**
4	432	745	**394**
8	622	968	**706**
16	947	1277	**1200**
32	1657	**2131**	2388
64	2913	**3745**	4426
